# Altered recognition of fearful and angry facial expressions in women with fibromyalgia syndrome: an experimental case–control study

**DOI:** 10.1038/s41598-022-25824-9

**Published:** 2022-12-13

**Authors:** Federica Scarpina, Ada Ghiggia, Giulia Vaioli, Giorgia Varallo, Paolo Capodaglio, Marco Arreghini, Gianluca Castelnuovo, Alessandro Mauro, Lorys Castelli

**Affiliations:** 1 “Rita Levi Montalcini” Department of Neurosciences, University of Turin, Via Cherasco, 15, Turin, Italy; 2 I.R.C.C.S. Istituto Auxologico Italiano, U.O. di Neurologia e Neuroriabilitazione, Ospedale San Giuseppe, Piancavallo (VCO), Italy; 3 Department of Life Sciences, University of Trieste, Via Edoardo Weiss 21, Trieste, Italy; 4 I.R.C.C.S. Istituto Auxologico Italiano, Laboratorio di Psicologia, Ospedale San Giuseppe, Piancavallo (VCO), Italy; 5 Dipartimento Di Psicologia, Università Cattolica del Sacro Cuore, Milan, Italy; 6 I.R.C.C.S. Istituto Auxologico Italiano, U.O. di Riabilitazione Osteoarticolare, Ospedale San Giuseppe, Piancavallo (VCO), Italy; 7 Department of Surgical Sciences, Physical and Rehabilitation Medicine, University of Turin, Turin, Italy; 8 Department of Psychology, University of Turin, Turin, Italy; 9 Department of Medicine and Surgery, University of Parma, Parma, Italy

**Keywords:** Human behaviour, Fibromyalgia

## Abstract

Evidence relative to facial emotion recognition and the role played by alexithymia in fibromyalgia syndrome is rare and heterogeneous. In this work, we investigated this ability in fibromyalgia investigating the implicit behaviour in the facial emotion recognition task, focusing on fear and anger. Twenty women with fibromyalgia and twenty healthy women as controls performed a facial emotion recognition of fearful and angry expressions. Their implicit behaviour was scored in accordance with the redundant target effect. The level of alexithymic traits through a standard psychological questionnaire and its effect on behavioral performance were also assessed. Participants affected by fibromyalgia reported a lower level of accuracy in recognizing fearful and angry expressions, in comparison with the controls. Crucially, such a difference was not explained by the different levels of alexithymic traits between groups. Our results agreed with some previous evidence suggesting an altered recognition of others’ emotional facial expressions in fibromyalgia syndrome. Considering the role of emotion recognition on social cognition and psychological well-being in fibromyalgia, we underlined the crucial role of emotional difficulties in the onset and maintenance of the symptoms life-span.

## Introduction

As pointed out in a recent review^1^, few studies^2–4^ had investigated facial emotion recognition in fibromyalgia syndrome, all reported a generalized difficulty in this ability^2–4^. However, the origin of such an impairment is far away to be clarified^1^. As a somatic symptom disorder^5^, fibromyalgia is mainly characterized by bodily symptoms, such as widespread musculoskeletal pain, muscle tenderness, and decreased pain-tactile threshold^6^. Therefore, why would facial emotion recognition, which is a crucial ability for efficient and satisfying social interactions^7^, be altered in this very body-related syndrome? Weiß and colleagues^4^ posed the fascinating hypothesis according which altered interoception (i.e., the sense of the internal state of the body;^7^) and the prolonged experience of pain, as typically observed in fibromyalgia, may affect emotional processing, and specifically the ability to detect and decode efficiently the bodily sensations associated to emotions. Moreover, evidence of functional and structural alterations in those cerebral areas crucially involved in emotional processing, and specifically the amygdala,^9–11^ as well as altered neurophysiological activity (in the components of P2 and N250) associated to facial emotion recognition^12^ have been reported in fibromyalgia. Furthermore, from a psychological perspective, adults with fibromyalgia show emotional dysregulations, such as altered emotional regulation, avoiding and suppression^13^, and lower emotional repair (i.e., the ability to regulate emotional states, enhancing the positive states and minimising those which are negative)^14,15^. Also, they experience interpersonal difficulties, especially in intimate relationships^16,17^, and showed difficulties in social cognition^2,18,19^ and emotional intelligence^20^ when tested with validated experimental tasks and maximum performance measures, respectively. Similarly, emotional distress and interpersonal difficulties have been described in juvenile-onset fibromyalgia with symptoms tending to persist into late adolescence or early adulthood^21,22^. Overall, all this evidence supports the hypothesis of an altered emotional recognition in fibromyalgia. However, some methodological criticisms in the previous studies exploring this hypothesis^1^ limit the data interpretation.

In the present study, we investigated facial emotion recognition^23,24^ in women with fibromyalgia. We asked our participants to respond as soon as possible as they recognized the emotional target, which was a human face with a fearful or angry expression. The task grounds on the well-established attentional psychophysical phenomenon reported in the visual domain known as *redundant target effect*^25^ applied to emotional stimuli^26,27^: according to it, people respond faster when two identical emotional targets, such as faces, are presented simultaneously rather than when presented alone; moreover, the competitive presence of a non-identical stimulus (i.e., another emotion) leads to a lower velocity in detecting the stimuli and a reduction of the level of accuracy. This effect, according which the behavioral performance is scored, is *implicit*: it refers to an automatic attentional psychophysical response in the visual domain^26–29^ and then it is not impacted by response biases related to higher cognitive process such as volition and self-judgement^23,24,30^, as instead traditionally observed in the explicit version of the facial emotion recognition task^2–4^.

Here, we tested independently the emotions of fear and anger. Prkachin and colleagues^31^ observed that in those psychosomatic syndromes characterized by higher expressions of alexithymic traits, included fibromyalgia, the processing of these two emotions is particularly difficult, enhancing interindividual conflicts. Multiple evidence suggests that alexythimia (i.e., an emotional trait characterized by difficulty in identifying and communicating feelings, altered emotional regulation, and an externally oriented thinking^32^) interferes with facial emotion recognition [i.e.,^33–40^], possibly jeopardizing an efficient social functioning^41^. Those studies investigating the somatosensory reactions to emotional expressions suggest that the altered facial emotion recognition in the case of higher level of alexithymia may be due to difficulties in the emotional embodiment^39^ or in the processing of the internal affective states^36^, underlying the bodily components of emotions. Instead, in the case of visual recognition of facial expressions, Starita and colleagues^40^ proposed that individuals with higher alexithymic traits may need more perceptual information to identify emotional (and specifically fearful) expressions. Crucially, anger and fear have a very significant social meaning. The recognition of the others’ expression of fear represents an alert of a possible external danger in the environment, suggesting the need of a defensive or escaping behaviors to guarantee own safety^42^. On the other hand, an efficient recognition of angry expressions allows individuals to modulate their behavior appropriately to guarantee their own safety^43^, when they are confronting themselves with others who express negative, and potentially threating, feelings^44^.

As done elsewhere^2–4^, we compared the performance of a group of women affected by fibromyalgia with the performance of a group of women free from any kind of pain, as controls. Any behavioral differences in the performance at the facial emotion recognition task between these groups might suggest an altered emotional processing, in agreement with the previous evidence in the field^2–4^. Nevertheless, in case of no difference between groups, we may suggest that the alterations in facial emotional recognition in fibromyalgia emerge only when the ability is tested according to an explicit behaviour [as done in^2–4^] rather than an implicit one (as done in the present study).

In this research, we also tested the role of alexithymia on the facial emotion recognition ability in our participants. Previous evidence about the role of alexythimic traits on facial emotion recognition in fibromyalgia is rare and heterogeneous: Di Tella and colleagues^2^ observed higher difficulties in recognizing angry facial expressions in the case of higher levels of alexithymia, in line with some observations in not clinical populations^33,34^. On the other hand, Weiß and colleagues^4^ suggested that alexithymic traits do not affect facial emotion recognition. Therefore, the questions are still completely open.

## Material and methods

This study was approved by the ethical committees of the Istituto Auxologico Italiano, IRCCS, Milan, Italy and Città della Salute e della Scienza Hospital, Turin, Italy. Subjects participated voluntarily; they gave informed written consent, were free to withdraw at will, and were naïve to the rationale of the experiment. Moreover, they received no compensation for participating in the experiment.

### Participants

Only right-handers were included in this study. We recruited women, since fibromyalgia is less reported in men^45–47^ at the admission at the involved Institutions. Women were included in the present study if they received a diagnosis of fibromyalgia^48^ made by rheumatologists who are experts in the field. Exclusion criteria were the presence or history of a neurological or a severe psychiatric disorder. As suggested^1^, we computed the body mass index in kg/m^2^. Moreover, we assessed the level of disability associated with the disease through the Italian version^49^ of the Fibromyalgia Impact Questionnaire—Revised Form (FIQ-R;^50^; internal consistency *α* = 0.94; item-to-total correlation between 0.41 and 0.78).

In line with other studies^2–4^, we included healthy women as controls from previous database relative to the same task^23,24^. Exclusion criteria were: diagnosis of fibromyalgia, rheumatic diseases or chronic pain; neurological or psychiatric disorder, including clinical depression and anxiety disorders; under medical treatments in the previous three months.

For all participants, we assessed the level of depressive symptoms the Beck Depression Inventory^51,52^; internal consistency *α* = 0.86, test–retest reliability *r* = 0.93) and current state of anxiety (i.e., trait scale; *α* = 0.90; test–retest reliability from 0.73 to 0.86), and the relatively stable aspects of “anxiety proneness,” (i.e., state scale; *α* = 0.93; test–retest reliability from 0.16 to 0.62) through the State–Trait Anxiety Inventory^53,54^.

### Level of alexithymia

The level of alexithymic traits was assessed using the Italian version^55^ of the Toronto Alexithymia Scale–20 (TAS-20; *α* = 0.81; test–retest reliability 0.77)^56^. It provides a total score and three sub-scores relative to difficulties in identifying feelings, difficulties in describing feelings, and externally-oriented thinking. Individuals indicated the extent to which they agreed or disagreed with each statement on a five-point Likert scale.

### The facial emotion recognition task

**Figure 1:**
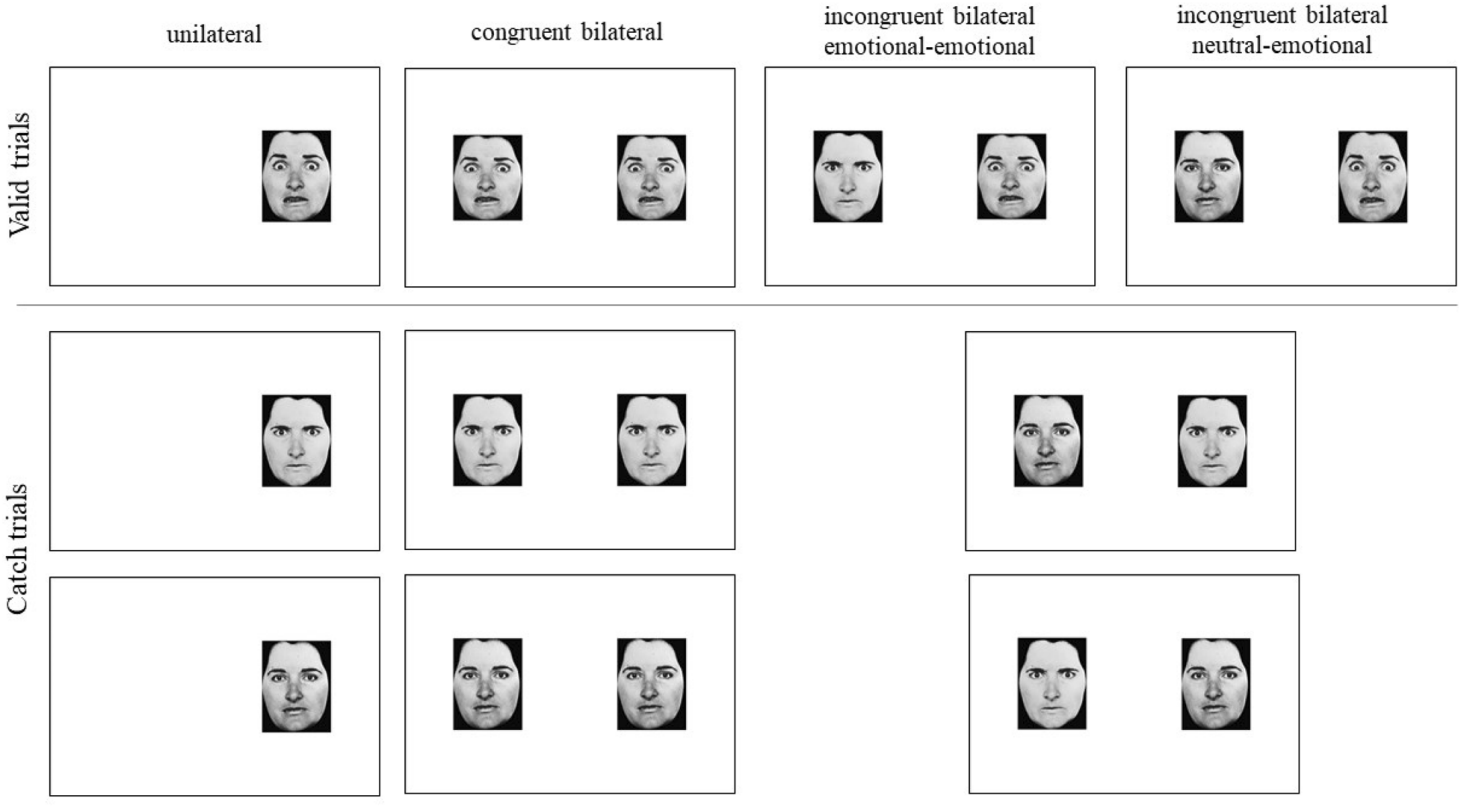
Experimental stimuli when the target was the emotion of fear, showed on the right part of the visual screen, for each of the four experimental conditions (congruent bilateral, incongruent emotional-emotional, incongruent neutral-emotional congruent). In the upper part, the stimuli for the valid trials are showed; in the lower part, for the catch trials.

We used a task described in previous studies^23,24^. It is a go/no-go task, in which photographs of male and female faces^57^ with either angry, fearful, or neutral expression, were presented on a screen in four different conditions: (i) in the *unilateral condition*, the target (anger/fear) was presented on the right *or* left of a fixation cross; (ii) in the *congruent bilateral condition*, the target was presented simultaneously on the right *and* left of the fixation cross; (iii) in the *incongruent neutral-emotional condition*, the emotion target was presented on the right *or* left of the fixation cross along with another but neutral face; (iv) in the *incongruent emotional-emotional condition*, the target was presented on the right *or* left of the fixation cross along with another emotional face (Fig. 1, upper part). The tasks consisted also of catch trials, in which the target emotion was never showed: a distractor (represented in half the trials by neutral stimuli, and in the other half by a contrasting emotion) was presented unilaterally in the unilateral condition, bilaterally in the congruent bilateral condition, or in opposition to a neutral/emotional stimulus in the incongruent conditions (Fig. 1, lower part).

Participants were instructed to respond as soon as they noticed the target (regardless of its position or number) on the screen, pushing a button on the keyboard with the dominant (right) hand.

Fear and anger were studied independently in different blocks. The target emotion was verbally announced by the experimenter at the beginning of each block. Stimuli stayed for a duration of 250 ms. Participants had a maximum of 1500 ms to provide an answer. The inter-stimulus interval varied randomly between 650 and 950 ms. For each condition (unilateral; congruent bilateral; incongruent emotional-emotional condition; incongruent neutral-emotional condition), 32 valid trials and 16 catch trials were presented in random order in four blocks. The block-order was reversely counterbalanced (i.e., ABBA order) to balance the order and sequence effects within subjects: half of the participants received the order ABBA: anger, fear, fear, anger; the other half, the opposite order BAAB: fear, anger, anger, fear. Overall, 768 trials were administered. There was a short break (two minutes) between each block. We assessed the percentage of *Accuracy* (% hits—% false alarms), which refers to the stimuli recognition. Moreover, the *Reaction Time* in milliseconds from stimuli onset was recorded relative to valid trials, which refers to the stimuli detection.

### Ethical approval

The study was conducted according to the principles of the Declaration of Helsinki. Patients gave their informed consent to participate.

## Analysis

### Descriptive characteristics

An independent sample t-test was used to assess any differences between the two groups relative to the demographical characteristics of age in years and education in years, the level of BMI, and the scores reported at the psychological questionnaires.

### The facial emotion recognition task

As done in previous studies^23,24^, reaction time and level of accuracy were first independently analyzed. Valid responses faster than 50 ms from stimulus onset were removed from the analysis since they were considered anticipations. A mixed ANOVA with the within-subjects’ factors of *Condition* (unilateral; congruent bilateral; incongruent emotional-emotional; incongruent neutral-emotional) and *Gender* (female vs male pictures), and the between-subjects factor of *Group* (participants with fibromyalgia vs controls) was performed. Bonferroni-estimated marginal mean comparisons were applied as post-hoc analyses when the main effect of *Condition* or the interactions were significant. In case of the significant main effect of the between-subjects *Group* or its significant interaction with the between-subjects factors, we planned to run the main analysis again introducing the global score at TAS-20 as covariate: this analysis allowed verifying if the main behavioral difference between our groups (if any) could be explained by the individual level of alexithymia.

Successively, we analyzed the speed-accuracy trade-off, computing the inverse efficiency score (IES) (the average of correct RTs divided by the proportion of correct responses^58^). When instructions require to be as fast as possible, as in our case, responding can become more error-prone if individuals are biased towards acting fast. IES allows to control for this trade off, and ensures relevant effects found are not explained by it. Thus, we used IES transformed data for a mixed ANOVA with the within-subjects’ factor of *Condition* (unilateral; congruent bilateral; incongruent emotional-emotional; incongruent neutral-emotional) and the between-subjects factor of *Group* (participants with fibromyalgia vs controls). Post hoc comparisons were carried out using estimated marginal means Bonferroni corrected for multiple comparisons, in case of significant interactions.

## A priori analysis

For this experiment, we a-priori planned to enroll a sample size of 20 participants with fibromyalgia to be matched with the 20 controls extracted from a previous database ^23,24^. Thus, we performed a sensitivity analysis to calculate the minimal statistically detectable effect size given a sample size of 40, and an a-priori level of power of 0.80 and an α was of 0.05 for the mixed (2 × 2x4) ANOVA. The effect size was of 0.12 with a critical F value of 1.68^58^.

## Results

### Descriptive characteristics and psychological questionnaires

Twenty women affected by fibromyalgia and twenty healthy women were enrolled. Means, standard deviations, and statistical results are reported in Table 1.

**Table 1:** Mean (M) and standard deviation (SD) relative to the demographical characteristics and the psychological questionnaires are reported for controls and the participants affected by fibromyalgia.

	Controls		Participants with fibromyalgia		Statistical analyses		
	*n* = 20		*n* = 20				
	M	SD	M	SD	t	*p* value	Cohen’s d
Age in years	47.9	11.56	47.75	12.66	0.03	0.09	< 0.001
Education in years	14.65	2.85	12.7	1.13	2.84	0.009*	0.89
Body mass index	22.26	1.24	22.05	2.05	0.38	0.7	0.01
Beck depression inventory	8	6	15	13	2.2	0.03*	0.69
**State–trait anxiety inventory**							
State-scale	35	9	37	9	− 0.48	0.62	0.22
Trait-scale	37	12	49	11	3.07	0.004*	1.04
**Toronto alexithymia scale (TAS-20)**							
Difficulty in identifying feelings	12.15	(4.21)	19.15	(8.05)	3.44	0.002*	1.08
Difficulty describing feelings	10.85	(4.18)	11.85	(4.4)	0.73	0.46	0.23
Externally oriented thinking	14.2	(6.18)	15.7	(4.66)	0.86	0.39	0.27
Total score	37.2	(12.38)	46.7	(14.06)	2.26	0.029*	0.71

Significant values (*p* value < 0.05) are in bold and indicate as*.

The two groups had comparable age and BMI, but different level of education. Participants with fibromyalgia reported significantly higher scores in the Beck Depression Inventory and in the trait-scale, but not in the state-scale measured with the STAI Questionnaire. At the Fibromyalgia Impact Questionnaire-Revised Form, participants with fibromyalgia reported the following scores: about functions, the mean was 17 (SD = 6; range = 6–27); overall impact, the mean was 9 (SD = 5; range = 2–20); symptoms, the mean was 32 (SD = 8; range = 17–47). Moreover, they reported a total score mean of 59 (SD = 17; range = 34–94), which suggested a medium (range 50–70) level of disability associated to the disease.

### Level of alexithymia

Means and standard deviations are reported in Table 1. Our participants with fibromyalgia reported a significantly higher total score in comparison with the controls, as expected. Specifically, they seemed to experience higher difficulties in identifying feelings, with no other difference.

### Facial emotion recognition task

Experimental data are shown in Table 2.

**Table 2:** Mean (M) and standard deviation (SD) for each experimental condition (congruent bilateral, incongruent emotional-emotional, incongruent neutral-emotional, unilateral) split for the visual stimuli gender (female vs male), relative to the two groups (participants with fibromyalgia vs controls) are reported about the reaction time (expressed in milliseconds) and the level of accuracy (expressed in percentage).

		Congruent Bilateral		Incongruent emotional-emotional		Incongruent neutral-emotional		Unilateral	
		Female	Male	Female	Male	Female	Male	Female	Male
**Fear**									
**Reaction time in ms**									
Participants with Fibromyalgia	M	413	413	428	439	455	419	435	419
	SD	192	159	185	202	160	146	180	126
Controls	M	368	354	429	411	418	394	372	366
	SD	117	94	124	143	132	93	87	92
**Accuracy in percentage**									
Participants with Fibromyalgia	M	45.89	55.49	21.63	30.45	20.94	28.92	50.31	58.33
	SD	19.45	19.64	20.10	19.24	23.57	16.83	19.13	19.00
Controls	M	63.52	66.51	37.57	48.58	39.51	49.83	61.98	69.32
	SD	19.38	14.77	22.35	16.34	22.56	17.63	18.36	12.95
**Anger**									
**Reaction time in ms**									
Participants with Fibromyalgia	M	399	394	384	439	418	436	420	414
	SD	128	156	142	187	141	154	138	155
Controls	M	374	386	410	423	407	430	375	405
	SD	98	149	84	166	102	123	103	160
**Accuracy in percentage**									
Participants with Fibromyalgia	M	40.40	43.16	22.29	22.29	15.21	17.43	54.03	44.44
	SD	28.11	21.09	14.23	13.70	21.22	15.96	22.70	21.18
Controls	M	59.57	55.61	41.28	32.53	33.92	29.76	62.07	56.25
	SD	18.76	*16.78*	17.12	20.85	17.34	23.02	17.15	19.59

The upper part regards the emotion of fear; the lower part, the emotion of anger.

### Fear

Because of the preprocessing data, the 0.11% of answers provided by the group of participants affected by fibromyalgia’s performance and the 0.99% provided by controls were not included in the analysis, since they were anticipations.

### Accuracy

**Figure 2:**
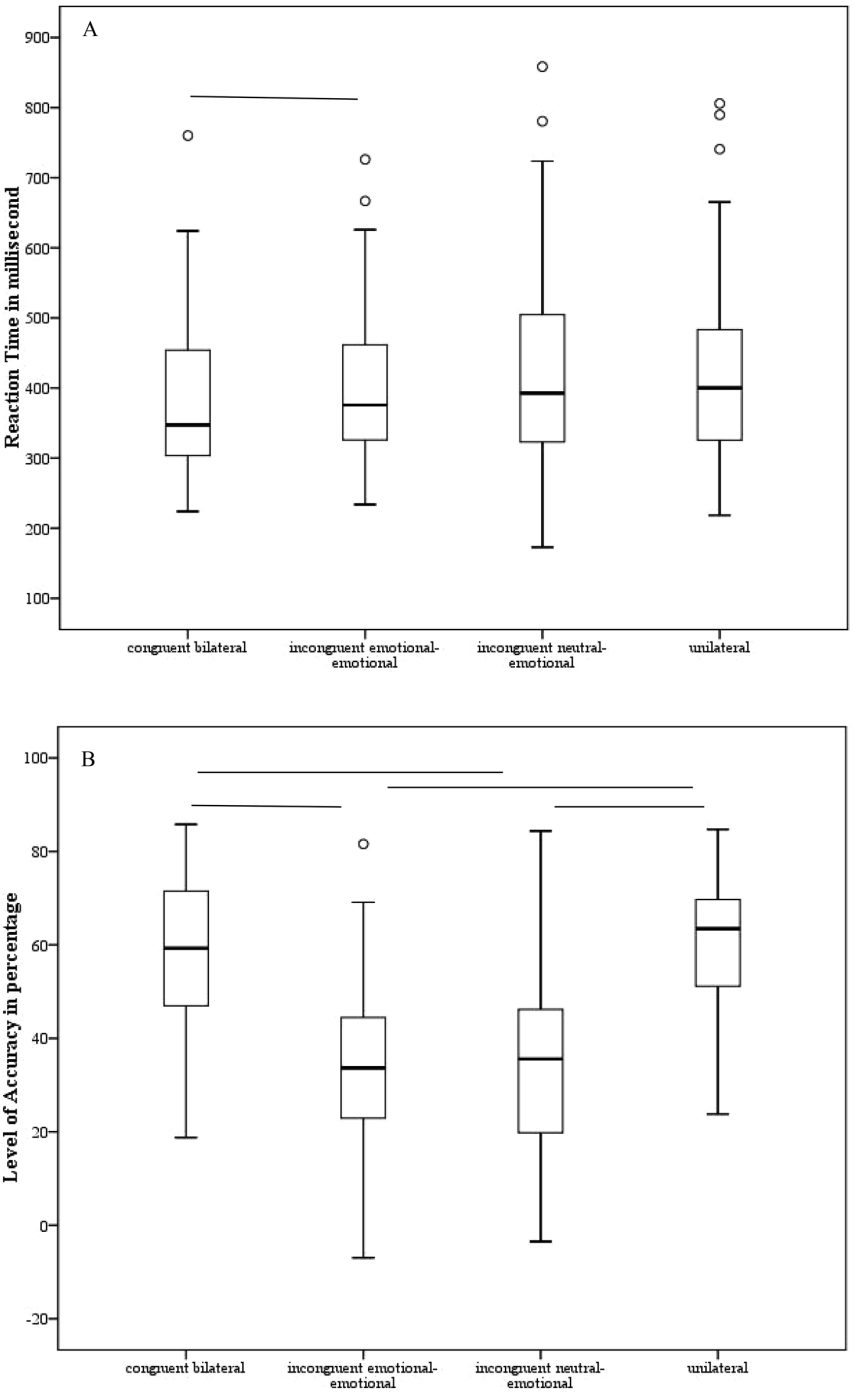
For each experimental condition (congruent bilateral, incongruent emotional-emotional, incongruent neutral-emotional congruent, unilateral; x-axis), the mean relative to the reaction time expressed in milliseconds (**A**) on the y-axis (left panels) and the level of accuracy (**B**) expressed in percentage on the y-axis (right panels) are depicted, when the emotion of fear was tested. The minimum, the lower quartile, the median, the upper quartile, the maximum, and the outliers are shown. Horizontal lines denote significant differences at *p* < 0.05.

A significant main effect of *Condition* [F(3,314) = 103; *p* < 0.001; η_p_^2^ = 0.73] was observed: all participants were significantly more accurate in the congruent bilateral and in the unilateral conditions in comparison with both the incongruent (neutral-emotional and emotional-emotional) conditions [p always < 0.001; upper part of Fig. 2—right panel]. This performance mirrored the experimental phenomenon of the redundant target effect.

**Figure 3:**
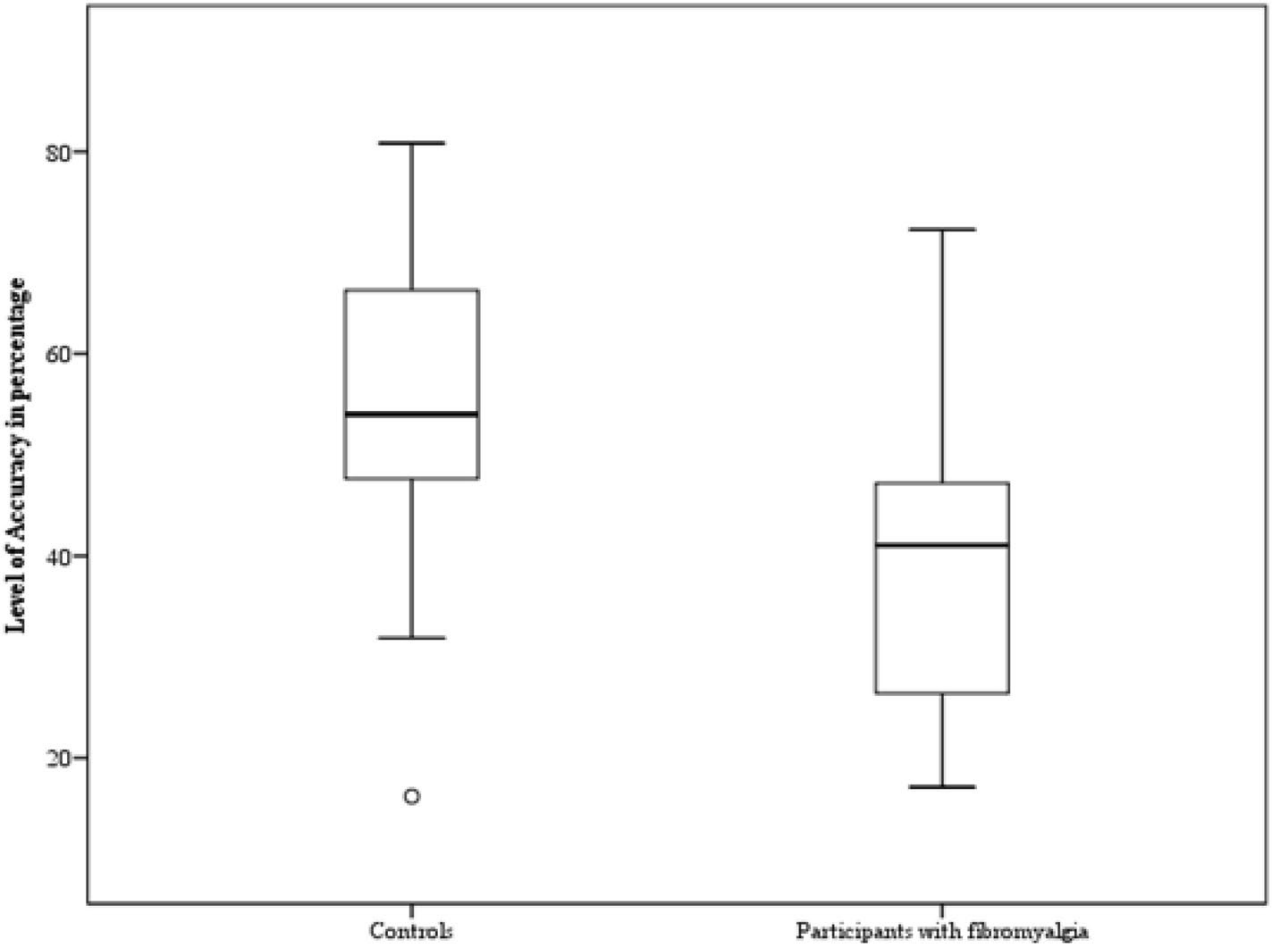
The mean relative to the level of accuracy expressed in percentage in the case of fearful expressions on the y-axis is shown for the two groups (controls vs participants with fibromyalgia). The minimum, the lower quartile, the median, the upper quartile, the maximum, and the outliers are shown.

We observed a significant main effect of *Gender* [F (1,38) = 14.4; *p* = 0.001; η_p_^2^ = 0.27]: participants were less accurate in recognizing fearful expression when showed by female faces (M = 42.66; SD = 2.86) than male faces (M = 50.92; SD = 2.26). Crucially, a significant main effect of *Group* emerged [F (1,38) = 11.11; *p* = 0.002; η_p_^2^ = 0.22]: as shown in Fig. 3, participants with fibromyalgia (M = 38.99; SD = 15.22) were significantly less accurate in comparison with the controls (M = 54.6; SD = 12.36). The first-level interactions [*p* ≥ 0.17] and the second-level interaction [*p* = 0.46] were not significant.

Since we observed a significant main effect of *Group*, we run again the repeated-measures ANOVA including the global score reported at the TAS-20 as a covariate. We confirmed the significant main effect of *Condition* [F(3,111) = 5.24; *p* = 0.002; η_p_^2^ = 0.124]. Interestingly, the main effect of *Group* still remained significant [F(1,37) = 7.09; *p* = 0.011; η_p_^2^ = 0.16]. The covariate [F(1,37) = 2.23; *p* = 0.14; η_p_^2^ = 0.05] as well as its interaction with the within-subjects factor of *Condition* [F(3,111) = 1.18; *p* = 0.31; η_p_^2^ = 0.03] were not significant. The interaction Condition*Group was not significant [F(3,111) = 1.25; *p* = 0.29; η_p_^2^ = 0.03]. Thus, the lower level of accuracy in recognizing fearful expressions observed in our participants with fibromyalgia was not relate to the higher levels of alexithymia.

### Reaction time

We observed a significant main effect of *Condition* [F (3,314) = 3.84; *p* = 0.01; η_p_^2^ = 0.09]: all participants were faster in the congruent bilateral condition in comparison with the incongruent emotional-emotional condition [*p* = 0.05], and with the incongruent neutral-emotional condition [*p* = 0.059] as a trend (upper part of Fig. 2—left panel). No significant main effect of *Gender* (female pictures M = 414; SD = 21; male pictures M = 401; SD = 18) [F (1,38) = 1.46; *p* = 0.23; η_p_^2^ = 0.03] was observed. No significant main effect of *Group* (participants with fibromyalgia M = 427; SD = 25; controls M = 389; SD = 27) [F (1,38) = 0.98; *p* = 0.32; η_p_^2^ = 0.002] emerged. Neither the first-level interactions [p ≥ 0.33] neither the second-level interaction [*p* = 0.74] were significant.

IES score. 3.2% of trial relative to the participants with fibromyalgia and 2.4% relative to the controls were excluded because out of the 2 standard deviations. The analysis confirmed the main effect of *Orientation* [F(1,33) = 17.14; *p* < 0.001; η^2^_p_ = 0.34]: IESs relative to the unilateral (M = 8.47; SD = 0.87) and the bilateral congruent (M = 7.9; SD = 0.59) conditions were similar [*p* = 1], whereas both were significantly lower in comparison with the incongruent bilateral emotional-emotional (M = 14.15; SD = 0.92) and neutral-emotional (M = 21; SD = 2.32) conditions [*p* always < 0.001]; moreover, the two incongruent conditions were significantly different [*p* = 0.01]: this pattern of results confirmed the presence of the redundant target effect. Moreover, the main effect of *Group* was significant [F(1,33) = 7.65; *p* = 0.009; η^2^_p_ = 0.85]: participants with fibromyalgia reported a higher score (M = 15.41; SD = 1.31) than controls (M = 10.35; SD = 1.27). The interaction was not significant [F(3,99) = 1.67; *p* = 0.17; η^2^_p_ = 0.04]. Overall, this analysis suggested that the difference observed in the main analyses between our groups was not related to a trade-off phenomenon.

### Anger

0.05% of answers relative to the controls’ performance was eliminated since they were anticipations. No data were excluded in the group of participants with fibromyalgia.

### Accuracy

**Figure 4:**
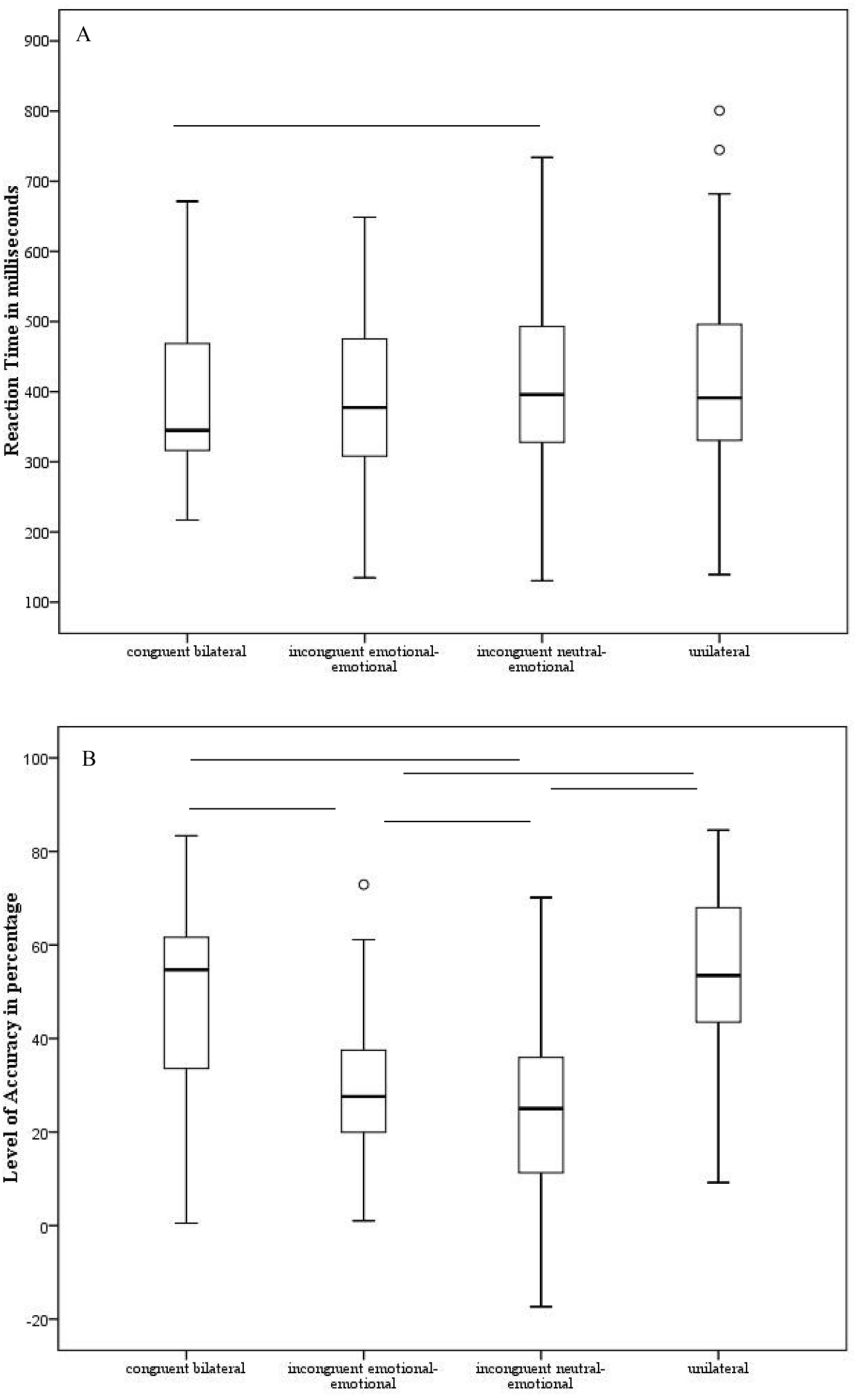
For each experimental condition (congruent bilateral, incongruent emotional-emotional, incongruent neutral-emotional congruent, unilateral; x-axis), the mean relative to the reaction time expressed in milliseconds (**A**) on the y-axis (left panels) and the level of accuracy expressed in percentage (**B**) on the y-axis (right panels) are depicted, when the emotion of anger was tested. The upper part regards the emotion of fear; the lower part, the emotion of anger. The minimum, the lower quartile, the median, the upper quartile, the maximum, and the outliers are shown. Horizontal lines denote significant differences at *p* < 0.05.

We observed a significant main effect of *Condition* [F(3,314) = 122.21; *p* < 0.001; η_p_^2^ = 0.76]: all participants were significantly more accurate in both the congruent bilateral and unilateral conditions in comparison with both the incongruent (neutral-emotional and emotional-emotional) conditions [p always < 0.001], in agreement with the redundant target effect (bottom part of Fig. 4—right panel).

**Figure 5:**
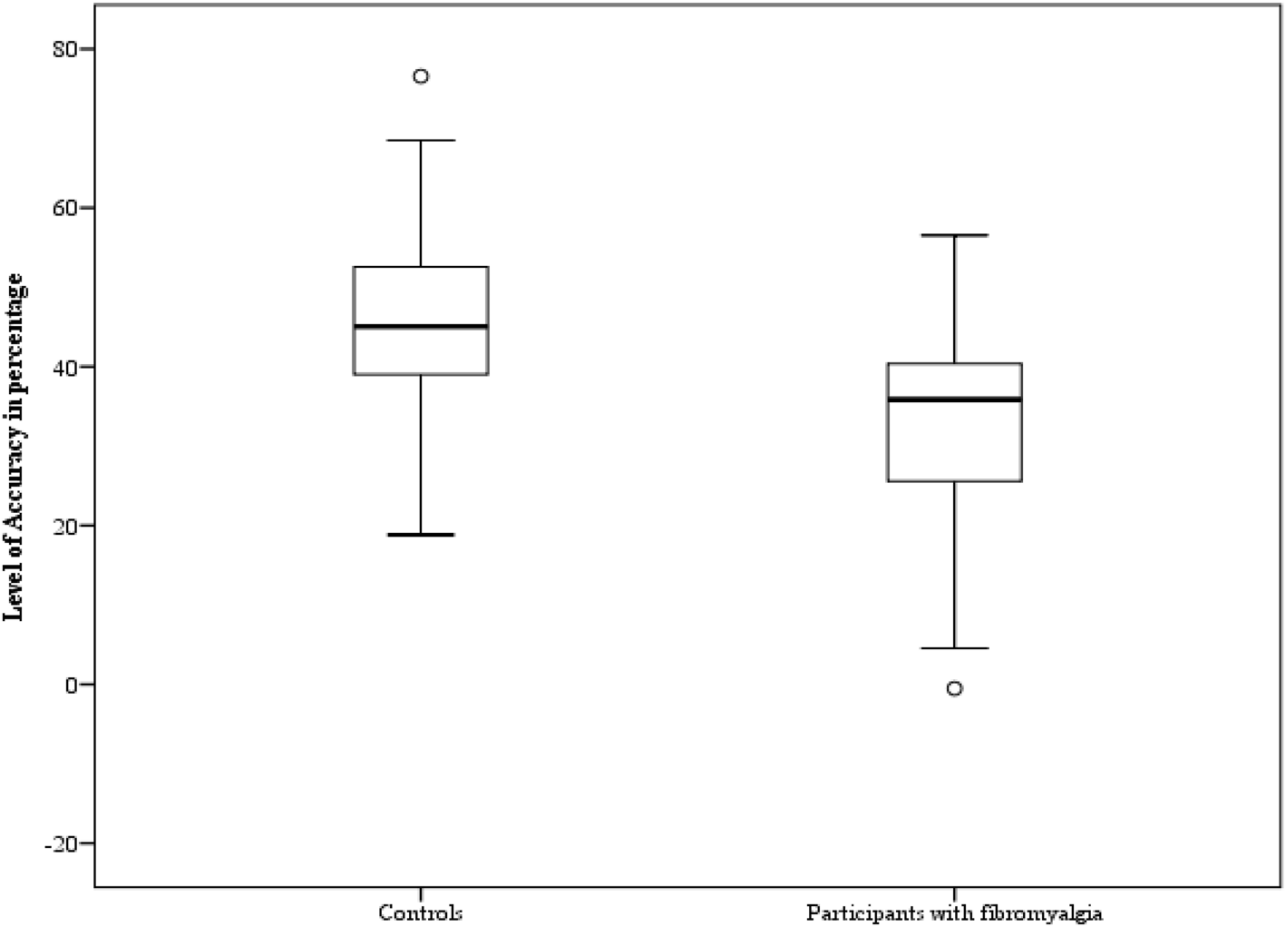
The mean relative to the level of accuracy expressed in percentage in the case of angry expressions on the y-axis is shown for the two groups (controls vs participants with fibromyalgia). The minimum, the lower quartile, the median, the upper quartile, the maximum, and the outliers are shown.

No significant main effect of *Gender* (female pictures M = 41.09; SD = 2.66; male pictures M = 37.68; SD = 2.76)[F(1,38) = 1.65; *p* = 0.2; η_p_^2^ = 0.04] was observed. Crucially, a significant main effect of *Group* emerged [F(1,38) = 8.71; *p* = 0.005; η_p_^2^ = 0.18]: participants with fibromyalgia (M = 32.4; SD = 4.86) were significantly less accurate than controls (M = 46.37; SD = 2.21) (Fig. 5).

Neither the first level interactions [*p* ≥ 0.09] neither the second level interaction [*p* = 0.19] were significant.

Because of the significant main effect of *Group*, we run again the repeated-measures ANOVA including the global score reported at the TAS-20 as a covariate. We confirmed the significant main effect of *Condition* [F (3,111) = 10.42; *p* < 0.001; η_p_^2^ = 0.22]. The main effect of *Group* still remained significant [F (1,37) = 6.85; *p* = 0.013; η_p_^2^ = 0.15]. Interestingly, the covariate was not significant [F (1,37) = 0.12; *p* = 0.73; η_p_^2^ = 0.003], as well as its interaction with the within-subjects factor of *Condition* [F(3,111) = 25.76; *p* = 0.35; η_p_^2^ = 0.78]. The interaction Condition*Group [F(3,111) = 1.24; *p* = 0.78; η_p_^2^ = 0.009] was not significant. Thus, the lower level of accuracy in recognizing angry expressions observed in our participants with fibromyalgia was not relate to the higher levels of alexithymia.

### Reaction time

The main effect of *Condition* [F(3,314) = 4.85; *p* = 0.03; η_p_^2^ = 0.11] was significant: all participants were significantly faster in the congruent bilateral condition in comparison with the incongruent neutral-emotional condition [*p* < 0.001]; moreover, a similar—even though no statistically significant—behavior was observed when comparing the reaction time relative to the congruent bilateral condition and the incongruent emotional-emotional condition [*p* = 0.058] (bottom part of Fig. 4—left panel). No significant main effect of *Gender* (female pictures M = 398; SD = 17; male pictures M = 415; SD = 23) [F(1,38) = 1.66; *p* = 0.2; η_p_^2^ = 0.04] was observed. No significant main effect of *Group* emerged (participants with fibromyalgia M = 413; SD = 17; controls M = 401; SD = 31) [F(1,38) = 0.9; *p* = 0.76; η_p_^2^ = 0.002]. Neither the first-level interactions [*p* ≥ 0.25] neither the second-level interaction [*p* = 0.09] were significant.

IES score. 2.4% of trial relative to the participants with fibromyalgia and 3.2% relative to the controls were excluded because out of the 2 standard deviations. The analysis did not confirm the main effect of *Condition* [F (1, 3) = 1.57; *p* = 0.02; η^2^_p_ = 0.04] (unilateral M = 7.52; SD = 0.65; bilateral congruent M = 7.02; SD = 0.55; incongruent bilateral emotional-emotional M = 11.31; SD = 2.47; incongruent neutral-emotional M = 12.19; SD = 3.36). Moreover, the main effect of *Group* was not significant [F (1,35) = 1.69; *p* = 0.2; η^2^_p_ = 0.04]: participants with fibromyalgia reported a IES (M = 10.98; SD = 1.62) that was similar to the controls (M = 8.04; SD = 1.57). The interaction was not significant [(F (1,105) = 0.01; *p* = 0.99; η^2^_p_ = 0.001]. Overall, this suggests that the difference observed in the main analyses between our groups would be related to a trade-off phenomenon.

### Supplementary analyses

The supplementary analyses (S1 in Supplementary Materials) confirmed that the results relative to the level of accuracy were not relate to the different level of education registered between our groups. As shown in Table 1, the two groups reported significant different scores at the psychological questionnaires relative to the depressive and trait-anxiety symptoms. Since, higher levels of emotional distress, such as anxiety and depression^60,61^, impact on facial emotion recognition, in the Supplementary Materials (S2), we reported further statistical analyses to explore if the level of accuracy would be explained by the different psychological functioning between groups. According to the results, the level of depressive or trait-anxiety symptoms did not explain the different level of accuracy registered in the main analyses.

## Discussion

We aimed to investigate the implicit recognition of fearful and angry facial expressions assessing an implicit behaviour^23,24^ and the role of alexithymic traits in fibromyalgia syndrome. Crucially, the previous evidence in the field is rare^2–4^ and all based on the assessment of the explicit behaviour at the task, which may be susceptible to methodological biases.

Our participants with fibromyalgia reported a significantly lower level of accuracy in recognizing facial expressions of fear and anger, when compared to free-pain controls. However, the performance relative to the recognition of the two emotions was not the same: the different performance observed between our groups about the emotion of fear was not related to a trade-off phenomenon, suggesting an overall difficulties of our women with fibromyalgia in recognizing efficiently facial expressions of fear. Instead, such a behavioral effect emerged for the emotion of anger: the difference between groups in term of accuracy may be significantly affected by the behavioral speed. This pattern of results recalls that fear and anger are two different primary emotions, and consequently they induce different behavioral reactions in humans, especially in social contexts. Specifically, in the case of fearful expressions, we tend to adopt defensive or escaping behaviors to guarantee own safety^42^; in the case of angry expressions, we tend to modulate our behavioral responses into the relationship with someone who is expressing a negative, and potentially threating, feeling^44^. This result, speaking in favor of a reduced ability in labelling correctly the facial emotion expressions, and especially about fearful expression, was in agreement with the previous experimental studies in which the explicit behaviour at the facial emotion recognition task was assessed^3,4^. Thus, such a difficulty seems to be independent from the level of awareness about the behavioral response implied by the experimental task, that was higher in Di Weiß and colleagues^4^, but lower in our experiment. According to this evidence, we may suggest a very pervasive effect of the disease on the emotional processing. However, focusing on the nature of the emotion, our results were only partially in agreement with Di Tella and colleagues ^2^, who reported a lower level of accuracy in recognizing the facial expressions of anger, but not of fear. Instead, we reported that such an alteration pertained both the emotions, with a possible interaction with the velocity in detecting efficiently the visual stimuli. The role played by top-down components^23,24^ on the experimental behavior cannot be excluded. For example, decision making about the nature of the emotion expressed by faces is largely involved in the case of the explicit behavior as assessed in Di Tella and colleagues’ study^2^, but it is less involved in the case of implicit behavior, as in this study.

Crucially, the lower level of accuracy registered in the performance of our participants with fibromyalgia was not attributable to the higher expression of alexithymic traits, at least in the case of an implicit behavior, independently from the emotion (anger or fear) tested. This result was in line with Weiß and colleagues^4^, who did not observe a relationship between the level of accuracy in recognizing emotional expressions and the level of alexithymia in their sample. Di Tella and colleagues^2^ reported no difference in recognizing basic emotions in a group of individuals with a higher expressions of alexythimic traits, when compared with a group with a lower expression, whereas they seemed to perceive angry expressions as expression of painful experience; however, it should important to underline that in this paper, the focus of the research was the attribution of pain (and not the primary recognition of emotions) on the facial expressions, altering possibly the results. Outside of fibromyalgia, the relationship between alexithymia and emotion recognition was verified, using a variety of different tasks^31,37,39^ with heterogeneous results. Thus, overall, the question about the influence of alexithymic traits on facial emotion recognition in fibromyalgia remains unsolved, requiring further investigation. Some other considerations can be done about our results. It isimportant to add that to assess the level of alexithymia, we used a very traditional clinical self-report^56^, as done in the field^1,62,63^. Our women with fibromyalgia reported specifically higher difficulties in identifying their own feelings, but not in the other components measured by the questionnaire^56^, which are the difficulties in describing feelings and the externally oriented thinking. Due to its features, this scale is an explicit assessment of the psychological functioning: respondents should be very aware of their reduced ability to identify and describe feelings in order to accurately describe such an alteration^23,64^. Thus, it may be argued that the scale does not measure the individual emotional capability, but rather its subjective description^65,66^. Following this consideration, the absence of any effect of the level of alexithymia on facial emotion recognition registered in our experiment may be related to the self-report nature of the questionnaire. Therefore, in case of higher levels of alexithymia (i.e., meaning the individual difficulties in recognizing or describing own emotions) as in fibromyalgia or in the case of not-clinical populations^35–40^, indirect measures of emotional processing should be used to avoid false-negative cases^23,34^: crucially, the behavioral (i.e., the experimental task) and psychological (i.e., the questionnaire) responses might not be strictly in the agreement with each other, as well as with the subjective emotional experience (i.e., the feeling)^67^. Moreover, even though alexithymic traits and facial emotion recognition are both components of the emotional processing, they pertain to two different dimensions: the psychological construct of alexithymia highlights the *intra-individual* dimension (i.e. how much I feel and express my emotions)^68–71^, although facial emotion recognition refers to the *inter-individual* dimension (i.e. the emotion expressed by the others). Since we observed that altered decoding of facial emotional expressions was not related to the self-report description of alexithymic traits, we underline the importance to adopt behavioral measures, as done in our experiment and in other studies investigating the physiological responses to the emotional stimuli [i.e.,^36,39,72^], together with self-report psychological assessments. For example, the somato-motor emotional processing should be explored in fibromyalgia, considering the evidence about its alteration in the case of a higher expression of alexithymic traits^39^; indeed, this observation might be crucial in defining the emotional experience in fibromyalgia, since it is conceived as a somatic symptom disorder^5^. Moreover, maximum performance measures as done in Luque-Reca and colleagues^20^ or validated measures of social cognition as done in Di Tella and colleagues^2^ may be also used to verify the emotional processing according to an implicit assessment.

Concentration difficulties, as part of the fibrofog,^73^ is commonly reported in fibromyalgia^74–77^ with possible negative side-effects on an efficient facial emotion recognition^2,3^. Because we did not include any neuropsychological measures in our experiment, we cannot exclude a priori the role of any cognitive alteration on our results. However, we underline that the redundant target effect is a psychophysiological automatic reaction generated by an external event (the stimuli), which acts on the level of attentional vigilance and behavior^28^. In our experiment, we registered this attentional phenomenon in our participants’ performance, suggesting that they have at least enough attentional resources to efficiently detect the emotional stimuli, even though the syndrome. In other words, we reported difficulties in decoding facial expressions in fibromyalgia, in presence of psychophysical answers which were coherent with the attentional mechanism. However, it should be noticed that our results relative to the reaction time were unique: indeed previous studies^2–4^ scored the performance only in terms of level of accuracy, but not of velocity, and then they did not take in account the trade-off phenomenon, as done in the present work.

We finally underlined some criticisms, which may be solved in future research. Due to the small sample size, our results may have been prone to type II errors, and we may have overinterpreted or misinterpreted our data. We tested only the recognition of fearful and angry expressions, since the evidence provided by Prkachin and colleagues^31^ relative a higher alteration of the processing of these two primary emotions in alexithymia. Nevertheless, if we would investigate multiple primary emotions in the same experiment, the amount of trial repetitions should be reduced as well as inferences about participants’ recognition can be done only about the global emotional processing, with no information about the single emotion^34^. Because of only two emotions were tested in this study, we cannot exclude that the difficulties registered about fear and anger here would be observed in the case of the other primary emotions, suggesting a global difficulty in the facial emotion recognition rather than an emotion-dependent alteration. Moreover, in this study we did not assess systematically the level of perceived pain, which instead could affect emotion recognition ^1^. When the emotion of fear (but not anger) was the target, a gender-effect on the level of accuracy was observed: our (all female) participants were less accurate in recognizing fearful expression when shown by female faces in comparison with male faces. This result was only partially in agreement with Scarpina and colleagues^23,24^ in which an effect of actor’s gender on facial emotion recognition was observed; however, both male and female participants were tested in this previous study. Instead, only females were tested in this study, as done in other studies^2–4^, since the larger prevalence of fibromyalgia in females. Of course, fibromyalgia may occur also in men^45–47^, but this syndrome may be differently experienced by males and female^78,79^. In addition, gender seems to play a role in emotional regulation and recognition^80–82^. Thus, the evidence from this study should be only carefully extended to males.

Individuals diagnosed with fibromyalgia report to have experienced a larger number of more severely negative life-events both in childhood/adolescence and in adulthood than healthy individuals^83,84^. Moreover, juvenile-onset fibromyalgia has also observed, even though under-investigated^21,22^, with no study about facial emotion recognition ability. Also, juvenile-onset fibromyalgia is associated with marked difficulties in psychosocial functioning and decreased quality of life, with symptoms tending to persist into late adolescence or early adulthood for the majority of affected individuals^21,22^. The present study, together with others^2–4^, suggested an altered facial emotion recognition ability in this syndrome. However, this study cannot draw any conclusion regarding the causal relationship between such an alteration and the development of fibromyalgia or the development of alexithymic traits. Nevertheless, considering that fibromyalgia is described in the different life-ages (in children, adolescents, and adults), research should consider to investigate this syndrome and its emotional functioning longitudinally. Humans' experience of emotion and comprehension of others’ affective states^85^ as well as emotion sensitivity, especially towards fear and anger^86^, change substantially across the lifespan: this observation has considerable implications for everyday functioning and the development of psychopathology across the lifespan. So, we strongly suggest to explore this topic in the context of fibromyalgia, also through behavioral and neuropsychological approaches: may this syndrome, with its constellation of bodily and emotional symptoms, have a longitudinal course? The question is totally open.

## Supplementary Information

Supplementary Information.

## Data Availability

The dataset generated and analysed during the current study is available in the Zenodo repository (10.5281/zenodo.7337239) on reasonable request.
