# Supplementary material for: Altered recognition of fearful and angry facial expressions in women with fibromyalgia syndrome: an experimental case–control study
Source: Sci Rep. 2022 Dec 13;12:21498. doi: 10.1038/s41598-022-25824-9 (PMC9747799; doi:10.1038/s41598-022-25824-9)
Supplement: Supplementary file 1 — Supplementary Information. [file 41598_2022_25824_MOESM1_ESM.docx]

**Title: Altered recognition of fearful and angry facial expressions in women with fibromyalgia syndrome: an experimental case-control study**

**SUPPLEMENTARY MATERIALS**

Federica Scarpina ^a,b^, Ada Ghiggia ^c*^, Giulia Vaioli ^b^ Giorgia Varallo ^d,e^, Paolo Capodaglio ^f,g^, Marco Arreghini ^f^, Gianluca Castelnuovo ^d,e^, Alessandro Mauro ^a,b^, Lorys Castelli ^h^.

**S1**. ***The role of education.*** As shown in Table 1, the two groups reported significant different level of education. To verify if the main results relative to the level of accuracy would be explained by the different psychological functioning between groups, we performed an ANOVA with the within-subjects factors of *Condition* (unilateral; congruent bilateral; incongruent emotional-emotional; incongruent neutral-emotional) and the between-subjects factor of *Group* (participants with fibromyalgia vs controls), including the level of education as covariate.

**Fear.** We confirmed the significant main effect of *Condition* [F(3,111)=5.24; p=0.002; η_p_^2^=0.12]. Interestingly, the main effect of *Group* still remained significant [F(1,37)=7.23; p=0.011; η_p_^2^=0.16]. The covariate [F(1,37)=0.58; p=0.44; η_p_^2^=0.01] as well as its interaction with the within-subjects factor of *Condition* [F(3,111)=0.65; p=0.58; η_p_^2^=0.01] were not significant. The interaction *Condition*Group* was not significant [F(3,111)=0.9; p=0.44; η_p_^2^=0.02].

**Anger.** We confirmed the significant main effect of *Condition* [F(3,111)=7.44; p<0.001; η_p_^2^=0.16]. The main effect of *Group* still remained significant [F(1,37)=6.23; p=0.017; η_p_^2^=0.14]. The covariate [F(1,37)=0.13; p=0.71; η_p_^2^=0.004] as well as its interaction with the within-subjects factor of *Condition* [F(3,111)=1.18; p=0.31; η_p_^2^=0.03] were not significant. The interaction *Condition*Group* was not significant [F(3,111)=0.74; p=0.52; η_p_^2^=0.02].

Overall, these supplementary analyses confirmed that the different level of accuracy between groups in recognizing facial expressions was not explained by the level of education.

S1. ***The role of depressive symptoms and anxiety.***

Here we report further statistical analyses to explore if the level of accuracy registered in the main analyses would be explained by the different psychological functioning (i.e., scores relative to the depressive and trait-anxiety symptoms) between groups. We performed a supplementary ANOVA with the within-subjects factors of *Condition* (unilateral; congruent bilateral; incongruent emotional-emotional; incongruent neutral-emotional) and the between-subjects factor of *Group* (participants with fibromyalgia vs controls), including the psychological scores as covariates.

**Fear.** When we consider the score relative to the depressive symptoms, we confirmed the significant main effect of *Condition* [F(3,111)=39.19; p<0.001; η_p_^2^=0.51]. Interestingly, the main effect of *Group* still remained significant [F(1,37)=9.46; p=0.004; η_p_^2^=0.2]. The covariate [F(1,37)=0.004; p=0.94; η_p_^2^<0.001] as well as its interaction with the within-subjects factor of *Condition* [F(3,111)=1.16; p=0.32; η_p_^2^=0.03] were not significant. The interaction *Condition*Group* was not significant [F(3,111)=1.68; p=0.17; η_p_^2^=0.04]. When we consider the score relative to trait-anxiety, we confirmed the significant main effect of *Condition* [F(3,111)=7.33; p<0.001; η_p_^2^=0.16]. Interestingly, the main effect of *Group* still remained significant [F(1,37)=6.87; p=0.013; η_p_^2^=0.15]. The covariate [F(1,37)=0.59; p=0.44; η_p_^2^=0.01] as well as its interaction with the within-subjects factor of Condition [F(3,111)=0.41; p=0.74; η_p_^2^=0.01] were not significant. The interaction *Condition*Group* was not significant [F(3,111)=1.59; p=0.19; η_p_^2^=0.04].

**Anger.** When the score relative to the depressive symptoms was taken into account, we confirmed the significant main effect of *Condition* [F(3,111)=45.77; p<0.001; η_p_^2^=0.55]. The main effect of *Group* still remained significant [F(1,37)=10.15; p=0.003; η_p_^2^=0.21]. The covariate [F(1,37)=1.36; p=0.25; η_p_^2^=0.03] as well as its interaction with the within-subjects factor of *Condition* [F(3,111)=0.41; p=0.74; η_p_^2^=0.01] were not significant. The interaction *Condition*Group* was not significant [F(3,111)=1.09; p=0.35; η_p_^2^=0.02]. When we took into account the score relative to trait-anxiety, we reported again the significant main effect of *Condition* [F(3,111)=4.63; p=0.004; η_p_^2^=0.11]. Crucially, the main effect of *Group* still remained significant [F(1,37)=10.55; p=0.002; η_p_^2^=0.22]. The covariate [F(1,37)=1.7; p=0.19; η_p_^2^=0.04] as well as its interaction with the within-subjects factor of *Condition* [F(3,111)=0.85; p=0.46; η_p_^2^=0.02] were not significant. The interaction *Condition*Group* was not significant [F(3,111)=0.85; p=0.46; η_p_^2^=0.02].

Overall, these supplementary results confirmed that the different between-groups level of accuracy registered in the main analyses was not linked to the different level of depressive or trait-anxiety symptoms.
